# Epigenetic Signatures in Hypertension

**DOI:** 10.3390/jpm13050787

**Published:** 2023-05-01

**Authors:** Gerardo Alfonso Perez, Victor Delgado Martinez

**Affiliations:** Facultad Medicina, Universidad Complutense Madrid, 28040 Madrid, Spain

**Keywords:** hypertensive, pre-hypertensive, machine learning

## Abstract

Clear epigenetic signatures were found in hypertensive and pre-hypertensive patients using DNA methylation data and neural networks in a classification algorithm. It is shown how by selecting an appropriate subset of CpGs it is possible to achieve a mean accuracy classification of 86% for distinguishing control and hypertensive (and pre-hypertensive) patients using only 2239 CpGs. Furthermore, it is also possible to obtain a statistically comparable model achieving an 83% mean accuracy using only 22 CpGs. Both of these approaches represent a substantial improvement over using the entire amount of available CpGs, which resulted in the neural network not generating accurate classifications. An optimization approach is followed to select the CpGs to be used as the base for a model distinguishing between hypertensive and pre-hypertensive individuals. It is shown that it is possible to find methylation signatures using machine learning techniques, which can be applied to distinguish between control (healthy) individuals, pre-hypertensive individuals and hypertensive individuals, illustrating an associated epigenetic impact. Identifying epigenetic signatures might lead to more targeted treatments for patients in the future.

## 1. Introduction

Hypertension is a major risk factor that can lead to cardiovascular and other diseases [1,2]. This has been known for decades. It is caused by a combination of genetic and environmental factors [3]. Although there is an extensive amount of literature analyzing hypertension, the exact underlying processes leading to hypertension remain not well understood, perhaps because of the above-mentioned complex combination of genetic and environmental factors that cause it. Some authors such as Messerli et al. [4] have estimated that the likelihood of an individual developing hypertension in an industrialized country during their life is approximately 90%, so it is an exceedingly common occurrence and a major contributor to mortality. Hypertension has also been identified by many authors as a major risk factor for COVID-19 [5,6,7] and for cognitive impairment [8,9]. As shown in work by Angeli et al. [10], there appears to be a complex process linking COVID-19 and hypertension. The increased mortality risk of hypertensive patients is also well documented [11,12,13,14]. Hypertension during pregnancy is estimated to result in complications in 5% to 7% of the cases, with a substantial maternal and fetal morbidity [15]. Although there are some well-defined treatment guidelines, some authors such as Ott and Schmieder [16] have mentioned that there remain discordant opinions in some treatment aspects. Institutions such as the Japanese Society of Hypertension, the British Society of Hypertension and the World Health Organization (WHO) have their own guidelines. Hypertension can be caused by multiple factors [17,18], of which obesity is among one of the more common [19,20,21]. There is ample research, see, for instance, Caminiti et al. [22], mentioning physical exercise as a component of potential treatments. Given the central role of diet [23,24,25] in hypertension and the changes in nutrition and lifestyles, it is likely that hypertension will be a major health concern in the coming decades.

There are epigenetic changes associated with hypertension but this is a process not yet well understood. There are some interesting articles, such as those by Stoll et al. [26] and Wang et al. [27], finding epigenetic links between hypertension and DNA methylation and other epigenetic changes such as histone modification. Wang et al. [27] highlighted the importance of understating DNA methylation in order to understand the mechanisms involved in hypertension.

Dasinger et al. [28] found a relationship between methylation levels and salt-sensitive hypertension. In a related work, Millis [29] mentioned that it is known that DNA methylation can limit nephron development and hence play a role in hypertension associated with high salt diets. Demura et al. [30] went a step further and concluded that there is a causal relationship between DNA methylation and the development of hypertension. DNA methylation can regulate the expression of genes and hence be related with multiple types of diseases. Fan et al. [31] mentioned that there is an association between aberrant methylation of the GCK gene and essential hypertension. Other genes such as ACE II, IFN-γ, AGTR 1, ADD1, SCNN1B and TLR2 have been identified in the literature [32] as playing a role in hypertension. It is likely that multiple genes, potentially interacting in a complex way, have a potential impact on hypertension. There are some pharmacoepigenetic analyses, such as the one carried out by Nuotio et al. [33], that have determined the responsiveness of four classes of antihypertensive drugs using DNA methylation data. This is a promising area of research in personalized medicine, demonstrated by the different responses to antihypertensive drugs by different patients. In an interesting article, Hishikawa et al. [34] analyzed DNA methylation patterns in patients with chronic kidney disease. According to the authors, currently, the primary causes of chronic kidney disease are hypertension and diabetes and they managed to find methylation signatures potentially usable as prognosis predictors.

The analysis of DNA methylation in the context of hypertension has received less attention than some other diseases, particularly cancer, with authors such as Han et al. [35] mentioning that the study of DNA methylation in the context of hypertension is still in its infancy. DNA methylation is an epigenetic change consisting of the addition of a methyl group to a cytosine [36], and it is typically analyzed in the context of CpG (a cytosine followed by a guanine in the same strand). This is a naturally occurring process associated with aging [37,38,39]. As previously mentioned, abnormal DNA methylation has been associated with several illnesses such as different types of cancer [40,41,42], schizophrenia [43,44,45] Huntington’s disease [46] and sepsis [47], among other illnesses. Nguyen et al. [48] used DNA methylation and machine learning techniques to detect high blood pressure, obtaining an accuracy of 69%.

One of the underlying assumptions in this paper is that there could exist a complex relationship between the DNA methylation levels present in a patient and that individual having hypertension. There is no obvious indication to suggest that this relationship between methylation levels and the presence of hypertension is linear, and hence non-linear models, such as artificial neural networks, are used as a modeling tool. One of the advantages of this type of approach is that it does not require having a detailed understanding of the underlying processes [49,50], which in principle could be extremely complex. In other words, the training phase [51,52,53] of the algorithm tries to identify these relationships without needing to specify them a priori. Given that there are hundreds of thousands of CpGs and that the relationship between the methylation levels and the presence of hypertension might be related to a combination of several CpGs, using traditional (linear) techniques might be rather challenging. Hence, in this type of nonlinear model it is important to select an appropriate number of input variables, which in this case is the number of CpGs selected as inputs. Otherwise, if the number of input variables is too high, it is likely that there could be some overfitting [54,55,56] in the model, which will likely translate into poor generalization power. Machine learning techniques are well known techniques and have been applied to the study of cardiopathies, see, for instance, work by Sivanandarajah et al. [57] and Kusunose et al. [58].

The main objective of this article is to show that there is a clear epigenetic impact, measured using DNA methylation, on patients suffering from hypertension and that such an epigenetic fingerprint can be detected using machine learning techniques such as neural networks [59,60,61]. Another objective is to show that the DNA methylation fingerprint of pre-hypertensive and hypertensive patients is distinguishable and different from control (healthy) patients and that machine learning tools are appropriate to carry out this type of analysis.

## 2. Materials and Methods

For each individual, there are n CpG methylation levels. These methylation levels range from 0 to 1. This information can be represented as a column vector (Equation (1)):

(1) $$X^{j}=\left(\begin{array}{c} \;x_{1}^{j}\\ x_{2}^{j}\\ \vdots \\ \;x_{n}^{j} \end{array}\right)$$

where the index *j* refers to patient *j*. It is also convenient to create a vector *Y* categorizing the patients (Equations (2) and (3)):

(2) $$Y=\{y^{1},y^{2},\cdots ,y^{n}\}$$

(3) $$y^{j}=\left\{0,1,2\right\}$$

The $y^{j}$ term can only take 3 values, with 0 denoting a control case while a value of 1 or 2 denotes a hypertensive of pre-hypertensive patient. In the first part of the analysis, the approach consists of differentiating the epigenetic fingerprints of control (healthy) individuals compared to non-control individuals (containing in this case both hypertensive and pre-hypertensive individuals). Therefore, in this case, $y^{j}$ is reduced to (4):

(4) $$y^{j}=\left\{0,1\right\}$$

with 0 indicating control and 1 indicating no control. It is also convenient to define a row vector (Equation (5)).

(5) $$X_{i}=\left\{x_{i}^{1},x_{i}^{2},x_{i}^{3},\cdots ,x_{i}^{m}\right\}$$

where $X_{i}$ is a vector containing the methylation information for the same CpG for all the individuals analyzed. As an example, in this notation, $x_{i}^{2}$ is the methylation level of CpG number *i* for patient number 2. As previously mentioned, this methylation value is a number between 0 and 1, representing 0% methylation and 100% methylation. By definition, the methylation data cannot take negative values or values above 1, which would indicate a methylation of more than 100%. As a data quality check, it is important to ensure that all the CpGs methylation data are actually within this range. Otherwise, it would be indicative of some experimental data gathering issues.

### 2.1. Data

The data were obtained from the GEO database with accession number GSE 193795 (publically available) [62]. There are 132 individuals, of which 44 are control (healthy) individuals, 44 are pre-hypertensive and 44 are hypertensive. There is the same number of male and female patients in each group. The age range for all the groups was from 50 to 65 years old. In the control group, the systolic and diastolic blood pressures were, respectively, less than or equal to 120 mmHg and 80 mmHg. The patients had no coronary or heart disease and had normal blood lipids (cholesterol less than 5.18 mmol/L and triglycerides less than 1.70 mmol/L). The BMI for the control group was 24.1 ± 2.5. In the hypertension group, the systolic and diastolic blood pressures were, respectively, greater than or equal to 160 mmHg and 110 mmHg or (after hypertension medication) greater than or equal to 140 mmHg and 90 mmHg, respectively. The BMI for the control group was 27.3 ± 3.2. In the prehypertension group, the systolic blood pressure was greater than or equal to 120 mmHg but less than or equal to 139 mmHg, and the diastolic blood pressure was greater than 80 mmHg but less than or equal to 89 mmHg. The patients in the prehypertension group had no cardiovascular diseases and a BMI of 25.3 ± 3.3. The patients in all three groups (control, hypertension and prehypertension) met these criteria for a period of 2 years before inclusion in the analysis [62]. For each individual, there were 223,945 CpG DNA methylation levels obtained from peripheral blood using the standard Illumina protocol. The methylation levels are expressed as a percentage, with a value of 1 indicating 100% methylation and a value of 0 indicating no methylation. The data were quality tested, with patients with more than 1% of the CPGS methylation data missing excluded from the analysis. No additional normalization of the data was carried out.

### 2.2. Preliminary Filtering

Technological advances have facilitated obtaining the methylation levels of large numbers of CpGs. A first intuitive step is to obtain an estimate for the average methylation level of an individual. This can be done by estimating the average methylation level across all the CpGs. For comparability purposes, this approach needs to be performed using the same CpGs. Recent technological advances have substantially increased the number of CpGs that can be analyzed in a rapid and reliable manner. The first generation of machines were able to analyze a few thousand CpGs. Currently, some of the most used machines can analyze hundreds of thousands of CpGs, while some of the most modern machines approach one million CpGs per patient. For comparability purposes, when carrying out this type of analysis, it is important to ensure that the average methylation level of the patient is estimated using the same CpGs per each patient.

As a preliminary filtering, the correlation between each CpG $\left(X_{i}\right)$ and the classification vector (Y) is obtained. The $n_{0}$ CpGs with the highest correlation are then selected as inputs for the model and the rest of the CpGs are discarded. This is done in order to reduce the dimensions of the data. In this way, we have reduced the initial *n* CpGs to $n_{0}$ with $n_{0}<n$. These $n_{0}$ CpGs are the input for the artificial neural network that is then trained to generate classification estimates. An excessive number of inputs in the artificial neural network might translate into overfitting and poor generalization. In this type of situation, the neural network finds spurious relationships in the training data during the training phase, which translates into inaccurate classification forecasts in the testing dataset. This is a common issue in situations in which the number of inputs is large compared to the number of cases. In most recent methylation datasets, there are hundreds of thousands of CpGs analyzed, while the number of patients typically is in the hundreds or (more unusually) in the thousands. The objective of the neural network is to generate accurate classifications in the testing dataset. The training phase was carried out with 75% of the data. The rest (25%) will be used as the testing data. The testing data are not used during the training phase. A 10 times cross-validation was carried out in every simulation. The reported accuracy and other metrics are the results of the testing data. The goodness of fit of the classification estimates is estimated by determining the accuracy and other metrics (see Equations (7)–(11)). Given the random initialization of the weights in the neural networks, the results can vary from simulation to simulation. Hence, β=100 simulations are carried out for each configuration and a confusion matrix (cm) is obtained for each of these simulations (Equation (6)).

(6) $$cm=\left(\begin{array}{ll} \alpha_{1} & \alpha_{2}\\ \alpha_{3} & \alpha_{4} \end{array}\right)$$

From this confusion matrix, it is easy to obtain the estimates of the goodness of fit metrics:

(7) $$Accuracy=\frac{\alpha_{1}+\alpha_{4}}{\alpha_{1}+\alpha_{2}+\alpha_{3}+\alpha_{4}}$$

(8) $$Sensitivity=\frac{\alpha_{1}}{\alpha_{1}+\alpha_{3}}$$

(9) $$Specificity=\frac{\alpha_{4}}{\alpha_{2}+\alpha_{4}}$$

(10) $$PPV=\frac{\alpha_{1}}{\alpha_{1}+\alpha_{2}}$$

(11) $$NPV=\frac{\alpha_{4}}{\alpha_{3}+\alpha_{4}}$$

The structure of the artificial neural network consists of one hidden layer with 50 artificial neurons. The calculations were carried out using Matlab 2016 and R 4.2.3.

### 2.3. Secondary Filtering

It is also interesting to determine whether it is possible to further reduce the number of CpGs while maintaining the accuracy of the classification forecasts. This was done using three different metrics (Equations (12)–(14)):

(12) $$Standard\;Deviation=S_{i}=\sqrt{\frac{\sum_{j=1}^{m}x_{i}^{j}-\bar{x}}{m-1}}$$

(13) $$Interquartile\;Range=I_{i}=Q_{75}-Q_{25}$$

where $Q_{75}$ and $Q_{25}$ are the 75th and 25th percentiles.

(14) $$Range=R_{i}=\frac{sup\left(x_{i}^{j}\right)-inf\left(x_{i}^{j}\right)}{sup\left(x_{i}^{j}\right)+inf\left(x_{i}^{j}\right)}$$

The $n_{1}$ CpGs with the highest values for each of these metrics were selected (generating three different datasets). These three datasets of CpGs were then used as inputs to the neural network, with each configuration simulated β=100 times, and the average goodness of fit values were estimated.

### 2.4. Optimization

It will be shown that for some applications, such as differentiating between hypertensive and pre-hypertensive patients, the approach shown in the previous section does not generate accurate results. Hence, an optimization approach was followed. The objective of this approach is to find a suitable combination of CpGs to use as the inputs for a nonlinear classification algorithm such as a neural network in order to generate accurate classification forecasts. Starting with all $n_{0}$ CpGs, the accuracy (Equation (7)) is estimated. Then, one CpG (randomly selected) is removed and the accuracy recalculated. If the accuracy improves from the previous step, then this new configuration is accepted. If not, then the previous configuration is accepted. The process is then repeated *q* times until the $q_{max}$ level is reached or a minimum accuracy $\left(A_{min}\right)$ is achieved. In this way, in each iteration the number of CpGs either decreases or remains unchanged, but the number does not increase. The algorithm is designed in this to try to reduce the risk of overfitting in the model. This is particularly important in this type of situation in which the number of inputs is large compared to the number of cases analyzed. Reducing the number of inputs, in principle, might help to reduce the risk of finding spurious relationships in the data not supported by the underlying biological processes. This risk is additionally limited by dividing the data into different sub-datasets.

Similarly to the previous section, the algorithm was applied to the training dataset which contained approximately 75% of the data, while the goodness of fit estimates, such as the accuracy of the model, were estimated with the testing dataset. The testing dataset was not used during the training phase. This is the standard approach followed when training an artificial neural network and it is carried out to try to minimize the risk of overfitting in the model.

## 3. Results

### 3.1. Base Line

**Figure 1:**
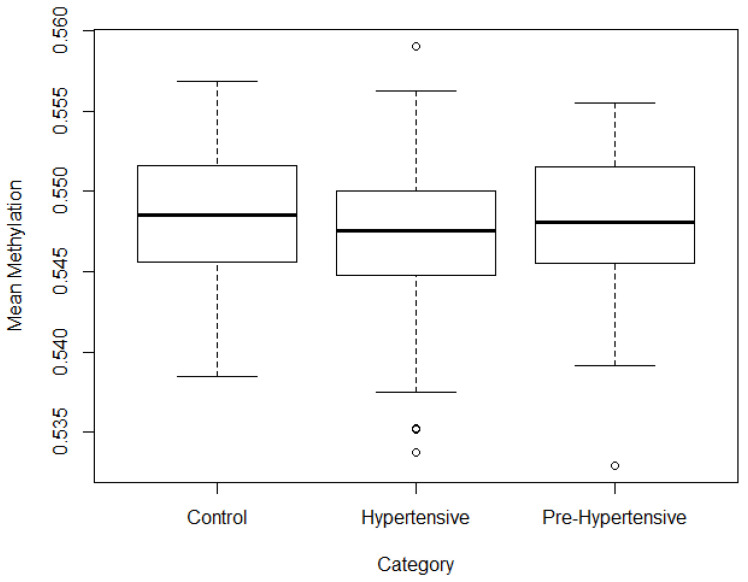
Average total methylation per category.

The process of estimating the average methylation levels was carried out separately for control, pre-hypertensive and hypertensive patients. The results can be seen in Figure 1. As it can be appreciated in the figure, there are no major overall methylation differences when comparing these three groups. The direct approach of a machine learning technique such as a neural network to the classification task of differentiating between control patients and hypertensive or pre-hypertensive patients, and hence identifying epigenetic signatures, does not generate accurate results. This is most likely due to the issues of overfitting and having too much noise in the input signal (too many CpGs for the model to efficiently identify appropriate patterns). There is hence the need to develop techniques that reduce the dimensions of the data, i.e., reduce the number of CpGs.

### 3.2. Filtering

**Figure 2:**
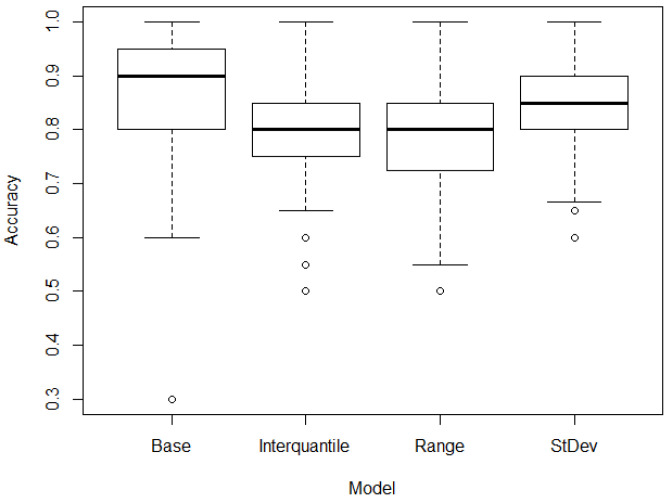
Accuracy (%) of the results of the base and further filtering models differentiating control and patients (including both hypertensive and pre-hypertensive).

A potential approach to overcome the issues shown in the previous subsection is to filter the CpGs. A preliminary filtering was carried out by obtaining the correlation between the methylation levels of each CpG and the categorical classification variable $Y=\{y_{1},y_{2},\ldots ,y_{n}\}$, with $y_{i}=\left\{0,1\right\}$ differentiating between control and patients. The top 1% of the CpGs was selected. This can be considered as a base case for the analysis, achieving accurate classification results as shown in Figure 2. It is also interesting to determine the effects of a substantial further reduction in the dimensions of the data (reducing the number of CpGs used). This was performed, as described in the Section 2, by estimating the metrics of interquartile, range and standard deviation. In this way, a further 99% reduction in the number of CpGs was achieved (selecting the top 1% of CpGs according to these metrics).

The results can be seen in Figure 2. Further filtering the data with these metrics shows that for the dataset analyzed, the accuracy results are statistically comparable (the numerical values can be seen in Table A1 in Appendix A), with the standard deviation metric generating the results with the highest median accuracy value. The mean accuracy for the base model was 86.3%. Other goodness of fit metrics can be found in Table 1. The model using the standard deviation as a filtering parameter achieved a goodness of fit statistically comparable to the base model, with a mean accuracy of 83.3%, using only the 22 CpGs that are shown in Table 2, compared to 2239 in the base model. The 2239 CpGs are shown in the Appendix A. The model with 2239 CpGs (base model) was obtained by filtering the DNA methylation data according to a correlation with the classification. The top 1% of the CpGs with the highest correlation (training dataset) was included in this model.

**Figure 3:**
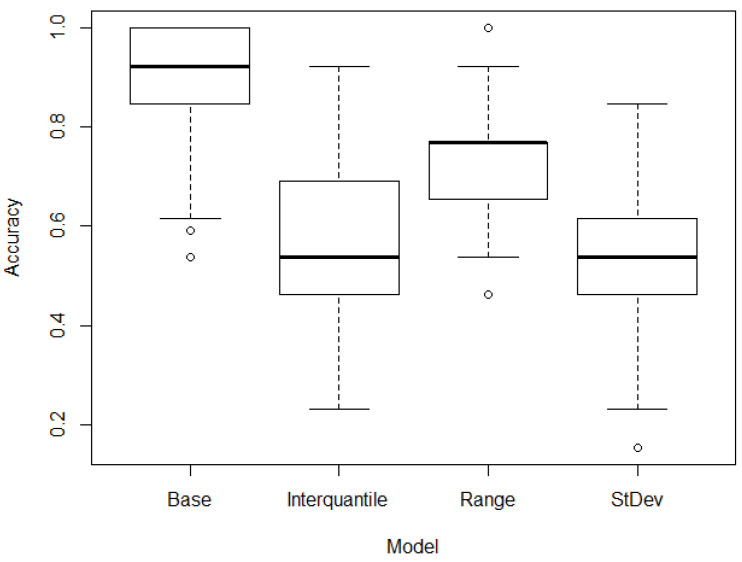
Accuracy (%) of the results of the base and further filtering models differentiating hypertensive and pre-hypertensive patients.

**Figure 4:**
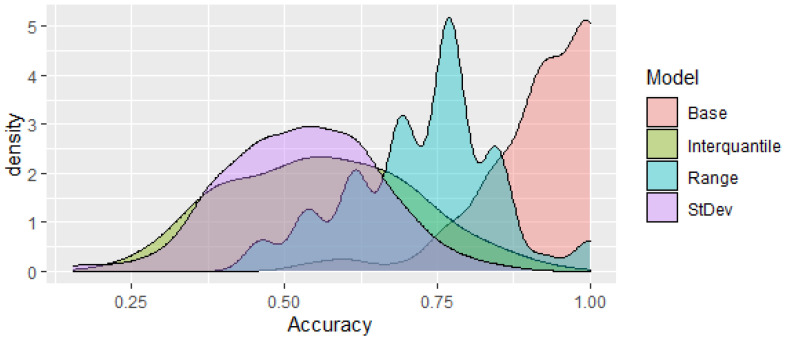
Density plots of the models based on interquartile, range and standard deviation metrics as well as the base model (accuracy %).

It is also important to differentiate not only between the control and patients, with the patient’s category including both hypertensive and pre-hypertensive patients, but also between hypertensive and pre-hypertensive individuals. The base case approach generated once more accurate results, but when the interquartile, range or standard deviation filtering was used, the accuracy did decrease in a statistically significant manner. A boxplot of the accuracy of these models differentiating hypertensive and pre-hypertensive patients can be seen in Figure 3. A density plot of the accuracy of the models can be seen in Figure 4.

### 3.3. Optimization

**Figure 5:**
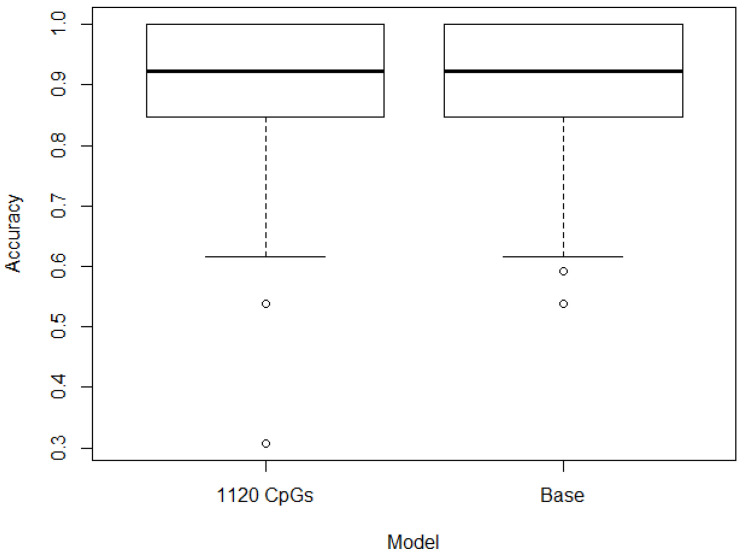
Accuracy (%) of the optimization algorithm (1120 CpGs) compared to the base case.

**Figure 6:**
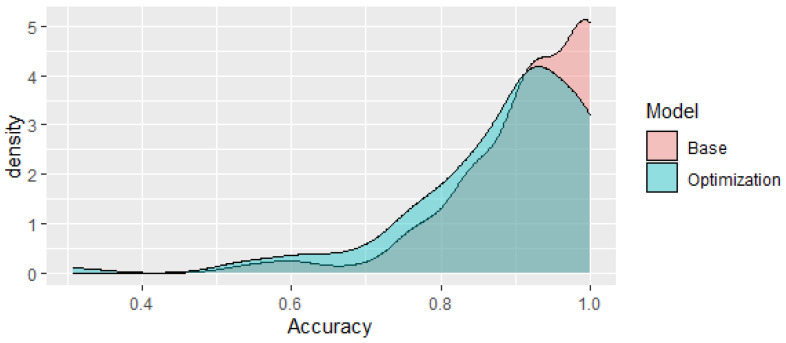
Density plots of the optimized and base models (accuracy %).

An optimization approach, as described in the Section 2, was then used to try to generate a model with a reduced number of CpGs that still generates comparable results. This optimization algorithm was able to reduce the number of CpGs to 1120 while obtaining an accuracy comparable to the base model (see Figure 5). The density plots of the accuracy of the optimization model and the base model are shown in Figure 6. It can be seen that they are a closer match than those using the interquartile, range and standard deviation metrics (Figure 4). The base case, using 2239 CpGs, generated a mean accuracy of approximately 91.9%, while the model using 1120 CpGs obtained a comparable mean accuracy of 88.3%. It is important to try to reduce the number of CpGs as a mitigation measure to limit the likelihood of overfitting the model and to avoid detecting spurious correlations with no biological link. Another important factor to take into account when creating this type of nonlinear model using machine learning techniques is not to make the models excessively complex with, for instance, an excessive number of neurons that could lead to poor generalization power when faced with new data.

## 4. Discussion

The analysis shows that there is a clear epigenetic signature associated with hypertensive (and pre-hypertensive) patients. These epigenetic signatures (abnormal DNA methylation levels) can be used to identify hypertensive patients with a relatively high level of precision. All the estimates of the goodness of fit of the classification forecasts were obtained using the testing dataset to minimize the risk of overfitting the models. This epigenetic signature was measured using DNA methylation levels of peripheral blood. Obtaining peripheral blood is a simple procedure, more convenient than obtaining cardiac tissue. This type of procedure could be used to screen for pre-hypertensive or hypertensive patients when they have a routine blood test. There is a large number of individuals with hypertension who are not aware of it. This type of test is unaffected by factors that could impact blood pressure levels in patients, such as stress or recent physical exercise, and can be used as an objective test to encourage the patient to adhere to a prescribed treatment.

Using a machine learning technique (an artificial neural network), it was possible to differentiate between control (healthy) patients and hypertensive or pre-hypertensive patients. It was possible to achieve an 86% level of accuracy using 2239 CpGs and a comparable 83% accuracy using only 22 CpGs. This accuracy is higher than some of the results published in the recent literature. It was also possible to differentiate between hypertensive and pre-hypertensive patients. Differentiating between hypertensive and pre-hypertensive patients was slightly more complicated, with the obtained model needing to use 1120 CpGs.

Nevertheless, it was possible to obtain a mean accuracy of 88.3%. This suggests that it might be possible to differentiate, following the same approach, between a more developed condition and a less developed condition. The accuracy obtained using these proposed approaches is higher that the results in some existing papers. For example, Nguyen et al. [48] obtained a 69% accuracy identifying hypertension. The results obtained in our analysis are consistent with the results in the existing literature, such as those presented by Dasinger et al. [28], Millis [29] and Demura et al. [30], suggesting that DNA methylation can be used as a tool to detect hypertension. As previously mentioned, this type of method could be used as an objective test (unaffected by external factors such as stress) to encourage the patient to adhere to treatment. Hypertension treatment typically requires lifestyle changes related to eating habits and exercise that some patients struggle to maintain in the long term. This type of analysis objectively shows the presence of the illness and can help convince the patient to maintain these lifestyle changes.

The analysis of abnormal DNA methylation associated with hypertension has attracted less research than similar analyses covering other illness, particularly cancer. Nevertheless, the results show that there are clear epigenetic signatures. This is a promising field. In the future, with more longitudinal data, it might be possible to use a personalized medicine approach to target more efficient treatments to patients using their DNA methylation profiles. This represents a field of promising future research, but there are also challenges. There is an increasing amount of DNA methylation data available, which is a positive development. However, these large amounts of information require sophisticated analyses. Given the apparent complexity of the underlying process linking DNA methylation with the presence of hypertension, it seems reasonable to use non-linear techniques, such as artificial neural networks. These techniques are prone to overfitting and hence special care is required to reduce the dimensions of the inputs, i.e., to do an appropriate filtering of the CpGs.

One of the drawbacks of using machine learning techniques such as artificial neural networks is that these models can become black boxes that are difficult to interpret. This is related to the idea that the relationship between the levels of methylation in a patient and the presence of hypertension might follow a nonlinear and complex relationship. In our case, this black box effect is mitigated because a significant part of the analysis targeted the reduction in the number of CpGs used as inputs, i.e., an input selection task. Particular care was also placed on not making the model excessively complex with, for instance, an excessive number of artificial neurons that could translate into low accuracy when faced with new data. Nevertheless, the complexity of analyzing this type of model, beyond the CpGs selection task, is acknowledged but is considered a reasonable trade-off when taking into account the high accuracy of these models.

## Figures and Tables

**Table 1:** Metrics for the goodness of fit of the base model.

Model	Mean
Accuracy	0.863
Sensitivity	0.727
Specificity	0.924
PPP	0.842
NPV	0.891

**Table 2:** List of 22 CpGs selected by the model (standard deviation filtering).

CpGs	CpGs	CpGs	CpGs
cg11538389	cg05410283	cg08937729	cg09853822
cg07352586	cg03077492	cg08213351	cg09163702
cg05879380	cg00026803	cg056612821	cg11791670
cg04966851	cg00316875	cg07400328	cg05650719
cg03531512	cg07991241	cg11175310	
cg02993069	cg00010992	cg11186962	

## Data Availability

The data were obtained from the GEO database. Accession code GSE193795.

## Appendix A

**Table A1:** Mean values and confidence interval for the base case and presented model.

Model	Mean	CI [95.0%]
Base model	0.863	[0.837 0.889]
Standard deviation model	0.833	[0.816 0.849]
Interquartile model	0.800	[0.779 0.820]
Range model	0.784	[0.764 0.804]
